# Ethical challenges for international collaborative research partnerships in the context of the Zika outbreak in the Dominican Republic: a qualitative case study

**DOI:** 10.1186/s12961-017-0246-0

**Published:** 2017-09-25

**Authors:** Julio Arturo Canario Guzmán, Roberto Espinal, Jeannette Báez, Ricardo Elias Melgen, Patricia Antonia Pérez Rosario, Eddys Rafael Mendoza

**Affiliations:** Centro Nacional de Investigaciones en Salud Materno Infantil Dr. Hugo Mendoza (CENISMI), Centro Los Héroes, Santo Domingo, República Dominicana

**Keywords:** Research ethics, Health equity, Health research systems, Capacity-building, Research networks, Zika virus, Disease outbreaks, Developing countries, Caribbean region, Dominican Republic

## Abstract

**Background:**

The establishment of international collaborative research partnerships in times of infectious disease outbreaks of international importance has been considered an ethical imperative. Frail health research systems in low- and middle-income countries can be an obstacle to achieve the goal of knowledge generation and the search for health equity before, during and after infectious disease outbreaks.

**Methods:**

A qualitative case study was conducted to identify the challenges and opportunities facing the Dominican Republic with regards to developing international collaborative research partnerships in the context of the Zika outbreak and its ethical implications. Researchers conducted 34 interviews (n = 30 individual; n = 4 group) with 39 participants (n = 23 males; n = 16 females) representing the government, universities, international donor agencies, non-governmental organisations, community-based organisations and medical societies, in two metropolitan cities.

**Results:**

Five international collaborative research projects related to the Zika virus were identified. Major ethical challenges were linked to the governance of health research, training of human resources, the institutionalisation of scientific activity, access to research funds and cultural aspects. Capacity-building was not necessarily a component of some partnership agreements. With few exceptions, local researchers were merely participating in data collection and less on defining the problem. Opportunities for collaborative work included the possibility of participation in international research consortiums through calls for proposals.

**Conclusions:**

The Dominican government and research stakeholders can contribute to the international response to the Zika virus through active participation in international collaborative research partnerships; however, public recognition of the need to embrace health research as part of public policy efforts is warranted. A working group led by the government and formed by national and international research stakeholders will be key to identify ways in which the country could respond to the ethical demand of generating new knowledge in times of outbreaks.

## Background

With the need to form international collaborative research partnerships in times of outbreaks, epidemics and pandemics have been considered an ethical imperative [1]. The weak development of national health research systems (NHRS) in low- and middle-income countries (LMICs) can undermine their ability to fulfil the international demand for knowledge production during and after public health emergencies. It is here that international collaborative research partnerships provide an opportunity to conduct the research that otherwise would not be implemented due to scarce resources, both human and material. Collaborative research projects could be helpful to strengthen local research capacities and reduce health disparities between and within countries [2–4].

We are in the midst of a new era where collaboration has become a standard of work between researchers, research centres and funders. There is now an account of the existence of a ‘science of collaboration’ with an ever-growing literature describing the conceptual basis, types, motivation for partnerships, and pros and cons of research collaborations [5, 6]. Those commenting favourably on research collaboration suggest that it allows the gaining of experience and benefiting from the abilities of others, it lowers the costs of scientific instrumentation, it provides access to information technology that facilitates professional exchange, and increases the quality of work and topic specialisation, among other advantages [5, 6]. Active involvement in cutting-edge, interesting science has been cited as a strong motivation for prestigious researchers to get involved in partnerships [7]. Conversely, the cited disadvantages include the metrics for co-authorships and the often negative connotations of the word ‘collaboration’ to indicate unfair relationships between North and South partners as well as unequal contracting policies, including data ownership, rights of authorships, access and control over funding, capacity asymmetries, and rewards and benefits of the partnership, among others. Dangers involved in international research collaborations have been identified elsewhere and are an important part of the research ethics literature in relation to informed consent, biobanking regulations, data sharing, benefit sharing, post-trial access, what constitutes a good or bad research collaboration, etc. [7–13].

To orient ethical international research, several widely accepted guidelines are in place [13–15]. The International Ethical Guidelines for Health-Related Research Involving Humans, specifically Guideline 8 on Collaborative partnerships, Capacity-building for Research and Research Review, states: “*The desired relationship is one of equal partners whose common aim is to develop a long-term collaboration through South–South and North–South cooperation that sustains site research capacity. To safeguard against power differences, innovative forms of collaboration should be considered*” [15]. Indeed, Emanuel [16] recognised the need for ‘collaborative research’ as an additional requirement when research is conducted by researchers of a high-income country in a developing country. Despite the wide acceptability of this ethical requirement, collaborative research is perceived as ethical imperialism or the exporting of research ethics principles to the global south [17, 18]. Hellman and Garrafa believe that collaborative research is a mere jargon utilised to induce “*passive acceptance of research projects as a result of rich (central) countries interest in peripheral countries*” [17].

Without any doubt, research may be required in times of epidemics due to the lack of a complete understanding of disease etiology, modes of transmission, prevention, detection, control or best treatment procedures. Collaboration may be the only way in which research could be performed at all, or at least in an efficient manner, due to the lack of expertise and the scarcity of resources (i.e. trained personnel, equipment, lack of technology) in the affected communities. The conduct of research during an emergency situation may prove to be rather challenging since humanitarian action predominates and knowledge generation may be perceived as a sideshow. These questions were especially relevant in the context of the Ebola epidemic, where the decision to conduct research in the middle of a public health emergency in a circumstance of human suffering with a life-threatening disease and social chaos was a source of conflict and disagreement [19]. Some challenges of the Ebola epidemic included the failure of international actors to act in a timely manner, the slow rate of funds reaching the field, the delay of ethics approval and the less than optimal data sharing practices [10–21].

These issues were the central theme of the 2015 Global Forum for Bioethics in Research (GFBR), a platform for the exchange and sharing of experience and expertise on research ethics among researchers, research policymakers and ethicists, among others. The GFBR aimed to explore ethical issues related to “*emerging epidemic infections and experimental medical treatments*” [22]. With delegates from over 35 countries, the forum presented pressing and unresolved ethical challenges when research initiatives occur during epidemics. Delegates were conscious that measures need to be taken before new epidemics appeared and lessons learned from the forum were drawn. In the same year, a cluster of microcephaly cases and other neurological disorders reported in Brazil led the Director-General of WHO to declare a Public Health Emergency of International Concern [23]. Research was considered crucial to reduce the uncertainty about the Zika virus and its consequences [1]. To support national governments and communities to prevent and manage Zika virus and its complications, WHO/PAHO and partners developed a strategic response plan that outlined four main recommendations, namely detection, prevention, care and support, and research [24]. Additionally, a virtual database was launched with active Zika studies to facilitate multidisciplinary research collaborations among scientists and a list of research articles and protocols [25, 26]. WHO’s international call for action brought challenges and opportunities for States, the international community with bilateral cooperation agencies, non-governmental organisations (NGOs), and the scientific community itself. However, LMICs that not have a functional NHRS in place may not be able to respond efficiently to this call for research in an emergency situation.

Therefore, concerned with the rise of the Zika outbreak in the Americas and with the interest to document how a developing country reacted to the outbreak in terms of research activities, we submitted a proposal for funding to the GFBR to support a small project looking to identify ethical challenges when developing collaborative research partnerships in the context of the Zika outbreak. The aim of this study is to explore the point of view of research stakeholders in the Dominican Republic regarding the challenges, strengths and opportunities for developing international collaborative research partnerships and their ethical implications. Other nations with similar socioeconomic characteristics as those of the Dominican Republic may also face these challenges. Findings from this study could provide useful insights about how to address these challenges in the Latin America and Caribbean region.

### The case under study

The Dominican Republic is a middle-income country with the largest economy in Central America and the Caribbean and high levels of growth in recent years, with a 7.3% growth in 2014. However, it also exhibits high levels of inequality according to the Gini coefficient (0.46) [26]. The Human Development Index is 0.722, occupying rank 99 out of 188 countries [27]. The maternal mortality ratio is 150 per 100,000 births, which is higher than the average of 130 per 100,000 in Latin America and the Caribbean, yet lower than the global average of 400 per 100,000 [28]. The infant mortality rate per 1000 live births is 26 [29].

Science and Technology (S&T) in the Dominican Republic have a recent history, beginning with a formal funding mechanism from the government through the National Fund for Science and Technology (FONDOCYT, by its acronym in Spanish) of the Ministry of Higher Education, Science and Technology (MESCyT). During 2005–2015, 335 projects were funded by FONDOCYT, of which 61 (18%) were destined to fund projects on biomedical research with a compromised amount of US$10,166,185 [30]. Most of the funding has been approved for research on biotechnology, genetics, food security, sustainable production, basic science and nanotechnology [31]. FONDOCYT does not provide funding for health research that does not include an innovative, technological or basic science component. International collaboration in S&T is incipient and possibly not well documented. The European Union Horizon 2020 Framework Programme for Research and Innovation, with initiatives such as the Network of the European Union Latin America and Caribbean Countries on Joint Innovation and Research Activities (ERANet LAC), has funded seven projects in 2015–2016, working in conjunction with 24 research groups from 14 European and Latin American countries [31].

Although health research is framed in the General Health Act as “*a basic and integral fundamental part of the process of health social production*” [30], minimal progress has been made in this area due to the negligible Ministry of Health (MoH) investment in health research. Therefore, significant gaps have been documented between what is researched and the population health needs, since health research funding is largely external and stems from private sources, such as the transnational pharmaceutical industry [32]. Health research collaborations are common and organised through universities and research centres, although no formal or systematic reports or public registry of such activities are available.

In terms of the response to the Zika outbreak, the MoH of the Dominican Republic adopted the recommendations issued by WHO. The MoH’s attention was directed almost exclusively to prevention and surveillance. At that time, no official sources confirmed research plans to be implemented regarding the Zika outbreak, either with national or international funding. Because a large proportion of studies in the Dominican Republic are externally funded and international calls for collaborative research in Zika were already in place, we anticipated that Zika research in the Dominican Republic would be funded by international sources. Therefore, more specifically, the questions addressed herein are, To what extent will the Dominican Republic design an agenda and allocate its own funding for this research? To what extent will Dominican researchers and institutions be able to apply or participate in international research? Which are the ethical challenges for those looking to implement international collaborative research?

## Methods

### Case study research design

This is a qualitative case study that aimed to reveal challenges for international collaborative research partnerships in the Dominican Republic, a Caribbean country of Latin America. We conducted in-depth interviews and focus groups with key informants to identify the challenges, strengths and opportunities of implementing international collaborative research partnerships. From the point of view of national research stakeholders, we sought to identify the stage of institutional research efforts and their willingness to engage in international research collaborations, subject to the mission and objective of each institution. The project activities, capacity-building in research and research ethics, and community engagement were then followed upon. We asked for recommendations about how to improve the health research system, as a manner to foster collaborative research partnerships.

### Characteristics of the investigators and reflexivity

Researchers were of Dominican origin, with expertise in public health, health research and international cooperation. The six-member research team included three physicians, one bioethicist, one clinical psychologist and one lawyer with expertise in international cooperation. Four researchers had a formal appointment at the Centro Nacional de Investigaciones en Salud Materno-Infantil Dr. Hugo Mendoza (CENISMI). Data analysis and interpretations reflected the vision of the research team and professional training in public health and research ethics.

### Sampling strategy

Through purposeful sampling [33], firstly considering the perspective of NHRS stakeholders, we listed sector, institutions, roles and positions conforming the population of the study. Therefore, from each institution, we selected the main official or its delegate and the sample size was determined by selecting one single participant from each previously identified organisation. Group interviews were performed when the institution asked for the inclusion of more than one participant. Table 1 shows the list of institutions and sectors considered for participation in the study.

**Table 1:** Distribution of interviews

Sector	Interviews (n)	Interviewed (n)		
		Fem	Male	Total
Dominican government	16	7	*11*	18
MoH	9	4	5	9
MEPyD	4	2	3	5
MESCyT	1	0	1	1
SISALRIL/SENASA	2	1	2	3
Universities, institutes and research centres	12	5	7	12
Public	7	4	3	7
Private	7	1	4	5
International cooperation agencies	3	2	4	6
Bilateral	1	1	2	3
Multilateral	2	1	2	3
ADARS	1	0	1	1
NGO’s	2	1	1	2
Total	34	15	23	39

*ADARS* Dominican Private Association of Health Management Organizations, *MEPyD* Ministry of Economy, Planning and Development, *MESCyT* Ministry of Higher Education, Science and Technology, *MoH* Ministry of Health, *NGO* non-governmental organisation, *SENASA* National Health Insurance, *SISALRIL* Superintendent of Health and Labor Risks

Letters of invitation containing information on objectives and procedures of the study were sent to the Director/President of the organisation, who indicated their representives in the study. After the initial contact, an appointment was scheduled for the key informant interview in a private office. In some cases, study participants provided referrals to other potential key informants. One institution officer declined to participate in the study, alluding to questions that undermined the confidentiality of private collaborative agreements.

Data source triangulation [34] was achieved by the inclusion of key informants coming from different sectors, such as research administration, policymaking and education, as participants and through the identification of policy brief documents, unpublished reports and publications on health research in the Dominican Republic. We asked participants to provide additional literature when possible. Additionally, we reviewed government websites, such as clinicaltrials.gov [35] and conabios.gob.do [36], to identify research studies that had submitted study protocols with pending or confirmed ethical approvals in the Dominican Republic. Researchers interviewed representatives with different institutional responsibilities as a way to complement information and strengthen the study validity.

### Review and ethical approval

The study protocol was reviewed and approved by the Research Ethics Committee (REC) of CENISMI in Santo Domingo, Dominican Republic. Participants provided verbal informed consent prior to participation in this study. Confidentiality was assured though the coding of participants and personal information was kept confidential. The recorded information was coded for analysis.

### Participants, methods and instruments of data collection

From August to November 2016, 30 semi-structured interviews and two focus group discussions were conducted with 39 participants (23 males, 16 females) in Santo Domingo and Santiago de los Caballeros, Dominican Republic. Each interview lasted approximately 60 minutes and was digitally recorded with prior authorisation of participants. Only one participant declined to be recorded, and field notes were taken.

An interview guide was designed to lead the discussion and allow flexibility for the presentation of new, salient issues to be included in the interview. To ensure the quality of information, an interview guide was developed for government, donor agencies and institutions conducting research (e.g. universities, institutes and research centres, NGOs). The questionnaire was composed of a total of 15 open-ended questions. Table 2 shows examples of questions included in the interview guide. The guidelines for conducting focus group discussions were adapted from the existing in-depth interview guides. Questions regarding experiences in research, collaborative projects on Zika and identification of the challenges, strengths and opportunities remained in all interviews and focus group guides.

**Table 2:** Interview guide: sample questions

Theme	Questions
Linking institution’s health research	How are the mission and mandate of your institution linked to health research?
	Does your institution have an explicit policy on research and an annual operating plan? Please, describe
	What are the funding sources identified for implementation?
Collaborative research	How do your mission, legal, strategic and/or activities support forming collaborative agreements for research development?
	Please describe the main achievements and progress made thus far
	Have these achievements been successful experiences of collaborative work?
	What are the main challenges that you face in supporting or sustaining collaborative research?
	Do you believe that there are any identified chances to develop partnerships for collaborative research?
Zika and research	What were the activities undertaken by your institution in the context of the outbreak of Zika?
	What are the main challenges in identifying opportunities of existing research?
Research capacities	What strategies do you consider that the Dominican government should take to strengthen research capacity?
	What strategies do you consider that the Dominican government should take to strengthen the ethical review process?
Community engagement	How should community participation be encouraged in project design, analysis and results?
	Could these programmes or projects involve and support vulnerable groups and minorities (e.g. women, disabled, sexual minorities, foreigners, low socioeconomic status)?
Recommendations for improving the steering and coordination of collaborative research efforts	What recommendations would you provide to improve the steering and coordination of collaborative health research efforts in the Dominican Republic?

Two focus group discussions were conducted. The first consisted of three NGOs with different profiles, including a community-based group working primarily with women, a hospital and a patient association (n = 3). The second was comprised of representatives from three specialised medical societies (infectology, gynaecology and obstetrics) and a university research institute (n = 4).

To guarantee the quality of the data during interviews, one researcher conducted the interview, while a second researcher facilitated the preparation of field notes regarding the context of the interview and drawbacks, such as frequent interruptions.

### Data analysis

Once compiled, all the interviews were uploaded to the qualitative analysis programme Dedoose to assist researchers in the data management during the coding process. Each interview was coded based on the topics delineated in the research objectives, including challenges, strengths and opportunities to develop collaborative research as well as strategies for capacity-building and community engagement. Data analysis continued until no new codes emerged, thus reaching saturation.

After coding, all excerpts related to the study question were searched and examined in unison. The lead investigator performed all initial coding, presented the code book to co-investigators, and coordinated a meeting to discuss the consistency in the application of these codes. Researchers performed a qualitative thematic content analysis [37, 38]. A first draft of the report was completed and discussed among researchers. The report followed guidelines for reporting qualitative research [39] and for key informant interviews and focus group discussions [40].

## Results

Our analysis resulted in five broad categories of challenges, and ethical implications, that can affect the possibility of implementing health research partnerships in the Dominican Republic (Box 1).

**Box 1:** Identified challenges pertaining to research response to the Zika epidemic in the Dominican Republic affecting its ability to join international collaborative research efforts

Governance of health research	
Institutionalisation and formalisation of scientific activity	
Trained and skilled human resources	
Access to research funds	
Cultural aspects affecting collaboration dynamics	

### Governance of health research

The Dominican Republic did not consider performing research as part of the national response to the Zika outbreak, even though foreign institutions were motivated to do so. One male MoH authority who participated in the Zika National Response Committee described the reactive and passive role of the MoH regarding research plans,

“*The reaction comes later on the part of North American universities that were very interested that the country develops a research agenda through the Ministry*” (MoH6).

Furthermore, senior MoH officials were unaware of the actual status of the MoH’s activities regarding research, claiming that they were not skilled in this area and that there were other managers who handled the investigation details. However, another MoH representative revealed,

“*The National Health Research Office was not involved, directly or indirectly, in the coordination of Zika response*” (MoH6).

Coordination, communication and collaboration among different Ministries were less than optimal. We set out to observe the level of cooperation, coordination and communication within the government in terms of efforts to initiate and conduct collaborative research partnerships. In this regard, a disarticulation of these efforts within the different instances of the Dominican Republic was noted.

A department of international cooperation from one office representing the Dominican government received an invitation from an Embassy to participate in an international call for application between researchers of their country and those in Latin America and the Caribbean. The participant said that they sent the information regarding the call for application directly to the MoH, and yet they could not identify someone at the MoH that was informed of this opportunity.

### The institutionalisation and formalisation of scientific activity

The institutionalisation of research in the Dominican Republic remains incipient in its organisations and in society as a whole. The labour of researchers will need a higher level of formalisation, including been recognised as a job in which people can make a living from it and having adequate technical and administrative support to manage research grants. In some universities, recruitment of researchers is based on ‘free’ hours of teaching. Additionally, the Dominican Republic MoH has not been able to establish a national health research institute and no official public registry of health researchers exists. Under these circumstances, participation in research projects comes with little prestige and with an additional work load, especially for those with clinical responsibilities where colleagues perceived a lack of motivation or loss of income in the case that they need to cancel appointments with private patients, as one participant pointed out. A participant also said that “*research infrastructure in the country is very limited*” (URC1). There is a lack of metadata on basic health indicators with limited access to scientific databases. Most of the universities do not have access to scientific databases.

### Trained and skilled human resources

A common subject identified as a challenge among participants was the lack of trained and skilled human resources in health research. For some of our participants, it is clear that the training is a prerequisite to good research and to be able to attract research collaborations. A Director of a private research institute stated,

“*It is unlikely to be selected as a partner in international collaborations when you do not have the required training and experience. Gaining experience requires investment and time with a forward purpose… The first thing that matters to a ‘partner’* [contributor] *is to see your CV, but if the ‘partner’ is not convinced by the expert or team, no pact, no alliance. A curriculum is built by a professional about five years after graduation. In this country, there is lack of policy and sustained investment…*” (URC10).

A lead researcher and authority in one university raised the issue of the lack of researchers and asymmetric capabilities,

“*It is necessary to level asymmetries in capabilities. It is a serious challenge, especially in health. Our doctors are very good doctors, but not good researchers. Because of their education, they are skilled to make a diagnosis and provide treatment. There are good researchers, but they are isolated cases*” (URC1)*.*

The participant continued, commenting that the profile of human resources dedicated to research must be more open and interdisciplinary:

“*Another error is a disciplinary perception in health issues. Health research is not an issue of doctors, but should include biologists, industrial engineers, anthropologists, sociologists, etc. So, we must demystify that health-related researchers are not necessarily medical doctors. Health, because of its complex nature, falls within what is known as transdisciplinary and, as such, the diversity of issues that are generated and new skills needed require an approach that goes beyond the traditional approach of the discipline*” (URC1).

The percentage of researchers in universities is minimal, less than 1% of professors are researchers and that reflects the relative scarcity of teachers with doctoral degrees or graduate research training. Training in research and bioethics is one of the areas that requires the most attention. Permanent programmes on research ethics were not identified as part of academic programmes. Medical societies do very little to provide training opportunities and accreditation of research competencies to their associate members, as expressed in the focus group discussions. Difficulties of local researchers at mastering English language skills is a common limitation. Patients and healthcare providers’ representatives recognised not having an active role advocating or supporting research initiatives.

### Funding

For some participants, the question of identifying funding sources represents one of the most serious challenges to conducting research during the Zika outbreak. Locally, no calls for research proposals in response to the outbreak of Zika were released by government research funding agencies such as FONDOCYT/MESCyT or the Fund for Economics and Social Research. The question of funding was interspersed with a lack of staff able to prepare fundable proposals on international calls. Two comments collected during interviews exemplify these challenges,

“*The challenges are mostly financial or about resources, also of staff that can quickly assemble the project…*” (URC6)

“*I think that here, as a country…the problem is not finding who will fund you. The problem is finding who can write the proposal*” (URC7)

### Cultural aspects affecting collaboration dynamics and motives to collaborate

Some participants commented on the motivation that leads to collaboration from all parties. Financial incentives and personal gain were not considered to be part of a sincere interest for collaboration. Two participants expressed these values:

“*What is important is the desire to do so* [the collaborative work]*, none of this creates a penny. People who are thinking about how to make more money are not interested in cooperation. Perhaps, it may be that at some point the cooperation can give you some benefit, but it is never a starting point of collaboration. I've noticed that when I talk to some people here, they do not feel encouraged to form a collaboration if there is not an economic incentive*” (URC10).

“*You need to be careful. There are some that come to us not because they are interested in the health of population but in their reputation*” (MS7)

The general idea of ‘transforming culture’ was collected systematically in several interviews and focus group discussions. A culture with the “*prevailing mentality or philosophy of ‘the separated store’ opposes the ethos of collaboration*”. This challenge entails the changes that must be made in moving the social values and practices that are part of a scarce culture of health research and of sharing scarce resources among locals.

### Strengths for the development of international collaborative research partnerships

The strengths for the development of research partnerships refer generally to the existence of the FONDOCYT/MESCyT. The FONDOCYT is a great stimulus for Dominican researchers in S&T. In its first edition in 2005, only four institutions of higher education participated in their call for funding. Later, in 2015, a total of 29 entities participated in the last call for funding, 17 of which were institutions of higher education [32]. Other strengths mentioned include the formation of research groups that are being created in universities and research centres, the establishment of competitive seed funding in some universities, and incentives awarded based on merit and scientific production.

### Opportunities for the development of international collaborative research associations

Outstanding opportunities have emerged in the Dominican Republic to perform collaborative work that includes the possibility of local researchers to participate in consortiums and in international calls for research and in scientific networks. One university participant stated that she knew of only one single opportunity to study Zika such as the Dominican Advanced Study and Research Network (RADEI) [41]. Other participants declared interactions with other scientific networks such as RedClara [42], Ibero-American Program of Science and Technology for Development (CYTED) [43], WHO Special Programme for Research and Training in Tropical Diseases [44] and EuraNet-LAC [45].

When WHO announced that a Public Health Emergency of International Concern was created by the Zika virus outbreak, several countries opened immediate international calls for research. A government official commented his experience connecting a foreigner with a local researcher,

“*A Canadian researcher interested in presenting joint research proposals made direct efforts to identify potential local collaborators in the* [Dominican Republic]*. MESCyT authorities, who in turn brought in local researchers from a university institute and a museum to conduct joint investigations. After several visits to the country, a funding proposal was submitted*” (DoG2).

Other participants noted the importance of recognition and prestige as researchers. Some highlighted that publishing in internationally prestigious journals was an opportunity for the development of collaborative projects. One participant mentioned that he found an international contact fortuitously through primary relations and not by the result of a predefined strategy:

“*You enter into the database of researchers so it is easier for people to call and meet you. At that first moment, we linked with a foreign university for primary relationships. You work with people, they know your job, and then they call you*” (NGO1)

Participants said that there is an ‘awakening’ to conduct research in the Dominican Republic. Since many actors are now interested in research, a formal body representing the various research stakeholders was suggested to contribute to the strengthening of the MoH stewardship function. One university official expressed his interest in collaborative work,

“*…We are totally open to collaboration. There is no research without that collaboration. We want to make agreements with several universities, private companies and the government*” (URC9)

### Research projects on Zika brought international collaborative partnerships into the Dominican Republic

In the Dominican Republic, very few researchers and institutions presented proposals for funding or finally implemented research in response to the Zika virus outbreak. A total of nine research projects on Zika virus were found through our interviews and search in the CONABIOS registry. Of those, five were international research projects (three in process of formalising agreements, one was active, and other concluded). Four small research projects were conducted by local researchers and funded by international cooperation agencies within larger prevention and surveillance initiatives. Table 3 summarises the research projects identified by our key informants. Only one study on Zika was listed in CONABIOS registry by September 2016.

We elaborate our report of the identified projects presenting four outstanding matters in research ethics.

**Table 3:** Research projects on Zika by institutions involved, study type – objectives and funding

Projects	Local institutions	External institution, country	Study type, objectives
Project 1	MoH/DIGEPI/Children’s Hospital	University, USA	Observational
Project 2	MoH/DIGEPI	University, USA	Observational
Project 3	Health centre (NGO)/Government	Government, USA	Vaccine clinical trial Phase II
Project 4	University	NGO Doctors of the World/CLACSO	Observational qualitative – literature review
Project 5	University	NGO Doctors of the World/CLACSO	Observational
Project 6	MoH/DIGEPI/Children’s Hospital/USAID	NA	Descriptive observational – analysis of the microcephaly case registry
Project 7	MoH/Directorate General of Epidemiology	NA	Observational – neurological damage and learning problems in children of women infected with Zika virus during pregnancy
Project 8	MoH/DIPRES	NA	Observational, KAP Survey on Zika High–Low Risk Communities
Project 9	Local NGO	NA	Observational, KAP survey in communities after an intervention

*CLACSO* Latin American Council Social Sciences, *DIGEPI* Directorate General of Epidemiology, *DIPRES* Directorate of Health Promotion and Prevention, *KAP* knowledge attitudes and practices, *MoH* Ministry of Health, *NGO* non-governmental organisation, *USA* United States of America, *USAID* United States Agency for International Development

#### Ethics review

The identified projects were at a very initial stage of agreements. None of the international studies was yet submitted for review to CONABIOS. By September 2016, only one study on Zika had been approved by CONABIOS. A double ethics review process exists; first, the protocol should be approved by an independent REC before the evaluation by CONABIOS. There are no uniform standard procedures available for RECs. We could not identify plans of RECs or CONABIOS to avoid long periods of review for protocols addressing public health emergencies.

#### Strategies for capacity-building in research and research ethics

From the interviews, we could not conclude that capacity-building was being considered as an integral part of research agreements. For example, university representatives commented that some research training activities were conducted in the context of postgraduate education although not related to specific research projects. Similarly, training in research ethics was not necessarily an integral part of partnership agreements. However, one participant mentioned that a research ethics committee was being set up in a regional public hospital with the support from an international research project.

#### Fair sharing of study responsibilities

A senior researcher and Director of one public health office within the MoH complained that participation and sharing of project responsibilities were not optimal. The protocol came already written in final versions, it had limited involvement in the identification and definition of the problem and selection of methodologies. She felt that this model of international research does not allow them to grow or develop their own skills.

#### Community engagement

In relation to community engagement efforts, resistance was noted. It is alleged that communities cannot give opinions on complex design issues in experimental studies. Participants mentioned that methodologies appropriate for that purpose are available, such as participatory and action-based approaches. Others felt uncomfortable using the term ‘community’ because it can be interpreted as vague. In general, the importance of sharing study results with the community was acknowledged.

## Discussion

Globalisation, the procurement of advancing science and efficiency considerations have led to an increment in international scientific collaborations; however, health equity has been brought to the discussion as a justifying principle to promote a shared governance of global health research consortia [46]. Still, we believe that NHRS have a duty to respond efficiently to public health emergencies of international concern, whilst doing their best to foster international collaborative research. NHRS should include the research mindset into national response activity and allocate resources from their own budget for this purpose. Achieving a holistic view of NHRS in LMICs is difficult due to a lack of real system functioning in practice; instead, we must consider how NHRS should work.

Challenges, strengths and opportunities for the development of international collaborative research partnerships in the context of the Zika outbreak in the Dominican Republic have been identified. Given the breadth of study results, our discussion focused primarily on the challenges encountered. Interestingly, this study attempted to observe the response of a Caribbean LMIC to the call of action for research during the Zika outbreak. The findings represent a systemic view of research as a function of NHRS that includes government and key stakeholders from academia, professional societies, NGOs and international cooperation agencies.

Although the Dominican Republic government implemented WHO recommendations in terms of prevention and surveillance of the outbreak, our findings suggest that research was not considered as part of the national response, thus failing to meet the demand for research in Zika. The very few projects were implemented were due to well-established researchers already working in stimulating environments with projects with previously known partners. This seems to indicate that collaborative spaces are not easily improvised in times of outbreaks, since establishing new alliances may require a longer time period than the duration of an outbreak. This individualistic approach is not sustainable; these lessons have already been learned during the Ebola epidemic – planning is basic. As stated by Horton et al. [5], “*Champions have a very limited function in partnerships – systems and structures are ultimately far more valuable*”.

WHO has proposed a functional, systemic and structural vision of NHRS that the Dominican Republic would do well to test [47]. From a NHRS approach, the State has a stewardship function which defines and articulates a vision of the system that includes priority setting development. Particularly in times of epidemics, emergencies and disasters, the role of governments in having an appropriate legal framework and in making available resources in response to the health emergency is emphasised [8]. In the words of Solimano [48],

“*The more developed countries, crossed by their own difficulties and by the conviction that effects are no longer confined to territorial borders, are urging third world nations to take the reins of their destiny and not expect everything from international support. Therefore, it is urgent to approach the issue from an innovative, collaborative, and participative perspective where resources, smaller every time, are better used*”.

International cooperation agencies are restricted to the programmatic agenda agreed with the government. If these themes regarding funding and scientific support during emergencies are not clearly delineated beforehand, then they will be oversighted due to the prominence of other public health activities perceived as more important and as having a higher immediate impact. In the present case, technical assistance and international cooperation provided by international cooperation agencies, including international NGOs, focused on supporting prevention measures in communities, detection of cases through laboratory tests and on the implementation of surveillance; research itself received little attention. It is important that States and international cooperation agencies jointly identify the opportunities for technical and financial assistance for health research in cases of outbreaks [49].

Challenges such as weak institutionalisation and formalisation of scientific activity have important ethical implications. As part of the NHRS function and responsibilities, if these problems remain unaddressed, then there is no guarantee that a nation can respond to the ethical commitment to contribute to the fight against infectious diseases through generating scientific knowledge. It is important to remark that, since our study data collection phase concluded, exceptional advances have been achieved in little time by the Office of Health Research of the MoH in the Dominican Republic – such efforts are encouraging.

Surprisingly for a small country, the Dominican Republic has more than 35 institutions of higher education, yet there is almost no formal training in health research, research methods or a Masters in Epidemiology with research concentration (Doctoral level). A formal programme on research ethics does not exist. Joint strategies should be sought between government and universities to train and maintain those involved in biomedical research up-to-date. Key elements in the training of researchers should include the promotion of stimulating environments, active identification of trainees, complimentary mentoring, and network consolidation [50, 51]. It is important to note that there are recently developed training resources specifically addressing ethics in epidemics, emergencies and disasters [49].

There are ethically questionable motivations to collaborations. Working with partners that are interested in increasing prestige, with no interest in population health, was seen as a threat to collaboration. However, this comment was in the general scope of collaborations and not specific of the current collaborative efforts in Zika. This idea of a ‘separated store’ also expresses an ‘ethos’, a way of being, where the desire for prominence, professional zeal, ambition to ‘earn more’, conflicts over intellectual property and authorship of publications characterise the ethical conflicts present in the scientific enterprise.

Five international research projects were identified, as well as four projects executed by local researchers that were funded by international cooperation agencies. However, at their initial stage of implementation, we identified common ethical issues that are well described in the research ethics literature. Participants alluded to their lack of involvement in the study design as researchers. The imbalance in the decisions about the research problem and research design, and being considered mostly as data collectors is a recurring theme in the international arena. A potential consequence of such practice is the imbalance in authorship. Furthermore, in projects where data-sharing and biobanking is required, an ethical challenge emerges when data and/or repositories are located in third countries other than the outbreak countries – there is a clear risk of disempowering the local research community, as one of our reviewers stated.

The ideal of establishing a real partnership based on reciprocity between the parties was discussed at the Forum, Beyond Aid … Research and Innovation as Key Factors for Health, Equity, and Development, held in South Africa in 2012 [48]. The Dominican Republic government, along with its main international partners, must work on establishing rooted partnerships. According to Yozwiak et al. [52], a ‘rooted partnership’ is based on the need to institutionalise research firmly in the affected countries. This vision of partnerships during public health emergencies incorporates four pillars, those of co-creation, capacity-building, sustainability and openness. Any form of collaborative research partnerships with these characteristics should be speedily promoted in the Dominican Republic to build trust and capacity to respond quickly and effectively to outbreaks, as well as to create a culture with transparent and inclusive measures that avoid ‘tourism research’ or ‘parachute researchers’ [52, 53], working from the beginning on the early formalisation of collaborative projects based on a Memorandum of Understanding [14]. Long-term relationships must be cultivated in the medium and long term [53].

Ethics review was given for one of the projects. We believe that observational studies should be reviewed by RECs. Since they are also prone to posing risks to study participants, such as psychosocial risks, valid informed consent processes, privacy and confidentiality should be evaluated. Other considerations might be added when dealing within emergency situations with pregnant women, children, elderly and prisoners as part of research subjects. RECs might also have a role in reviewing international research collaboration agreements. The fact that a project is conceived as an international collaboration does not indicate that it will conform to the ethical perspective of ‘collaborative research’. In their report, RECs could elaborate whether the project achieves the aims of a truly collaborative project, including answering questioning whether the project conforms to sharing data, recognition of local authorship, capacity-building activities, sharing benefits, local authorities represented, and fair and transparent research contracting policies and practices. RECs should identify minimum requirements to consider valid and acceptable collaborative agreements and their demands should be proportionate to the type, scope and funding of the project [14]. For example, RECs of institutions developing guidelines to review biobanking practices with data and/or sample repositories may send these for further analysis in to other countries, making local communities more vulnerable and disempowered, as stated by our reviewers. Checklists to evaluate the governance of research consortia [46] and questions to address shared knowledge and tools beyond research consortia are existent [54]. This kind of ethics review requires additional expertise and time, which may not be available in the REC at this present time. Funders also have a say when proposing and evaluating research consortia plans [55]. RECs should be trained and become familiar with critical elements such as benefit-sharing in international research and community engagement aspects. This kind of training should be performed both at the national and regional levels to ensure a common approach and understanding of the subject matter by the different RECs. Increased collaboration through REC networks is warranted. Aarons [56] presented a model for efficient coordination and communication between and among RECs and templates for expediting ethics review in emergency situations. Ideas like this one to aid REC collaboration and increased efficiency should be considered before new emergencies arrive in our doorstep. The strengthening of the institutional basis of RECs is also a pending task. An emergent Central America and Caribbean Network of Research Ethics Committees could address these issues in the region.

Finally, it was observed that, in academia and research centres, community engagement continues to be devalued in all phases of the study. Promoting the participation of civil society and communities is a challenge and an ongoing task. The recently revised version of Council for International Organizations of Medical Sciences Guidelines emphasises the need to be more transparent and to involve communities from study planning to implementation [14].

### Limitations

The ethical challenges of this study can be linked to initial stages of agreement formation that occur prior to the implementation of research, and not to trigger conflicts that may arise during or at the end of the project. At the same time, the issues discussed do not necessarily reveal the difficulties of those identified research projects, but rather represent the interviewee’s reflections on the ethical issues on international research partnerships in general. Some researchers were reluctant to answer questions about studies that were in phases of creating agreements. After we conducted the case analysis regarding the Dominican Republic, we believe that our findings are valid and apply equally to most of the countries in the Central America and Caribbean region.

## Conclusions

There is a definite ethical challenge in international collaborative research in times of epidemics with regards to the implementation of an actual research project or consortia that conform to an ethical framework that considers well-established ethical standards and health equity as an oriented principle. Research collaboration should not be interpreted as a ‘new way to conduct research overseas’ or just as a transactional activity or as a passive inducement to filter a research agenda. States, funders, researchers and RECs should have a shared responsibility towards collaborative efforts, particularly in emergency situations. States should consider addressing all aspects of well-driven national and international responses to public health emergencies.

Despite our focus on the importance of research to public health emergencies, we do not embrace the idea that research is the panacea to solve such problems, or that it should gather the majority of resources, rather that it should be used as a tool to learn how to better protect population health.

The Dominican Republic has the responsibility to contribute to the international response to the Zika virus, not only by using traditional public health prevention measures such as community-based advertising and prevention campaigns to eliminate vector breeding sites, but also through developing its own research agenda and planning its involvement in international collaborative research partnerships. This could be achieved through the development of its NHRS. It is encouraging that government officials, representatives from universities, private health associations and donor agencies expressed interest in generating collaborative agreements with local and foreign organisations. A working group, led by the government and formed by national and international research stakeholders, will be key to identifying ways in which the country could best respond to the ethical demands of operationalising and learning from infectious disease outbreaks of international importance. Local and regional RECs should carefully determine how to evaluate and dictate research collaborations from an ethics standpoint.

## Abbreviations

GFBR: Global Forum on Bioethics in Research
LMICs: Low- and middle-income countries
MEPyD: Ministry of Economy, Planning and Development
MESCyT: Ministry of Higher Education, Science and Technology
MoH: Ministry of Health
NGO: non-governmental organisation
NHRS: national health research systems
REC: research ethics committee
S&T: science and technology
